# Statement of a Case

**Published:** 1877-05

**Authors:** 


### "STATEMENT OF A CASE."

"The plaintiff, a man of about fifty years of age in apparently perfect health; without any discoverable syphilitic, scrofulous, scorbutic or other constitutional taint, and who had never been sick in his life and never had scrofula or syphilis ; has toothache in the lower wisdom tooth on the right side about one hour; goes to a dentist, (defendant) who attempts to pull the aching tooth, using Physick's elevating forceps, he breaks off the crown leaving the fangs in the socket ; in few hours the jaw begins to swell, and within a day or two patient is unable to open his mouth at all. Violent ostitis sets in and the whole jaw bone becomes involved; fistulous abcesses form under the jaw and large quantities of pus are discharged through these abcesses and through openings made by lancing for that purpose. The patient for ten weeks, beginning immediately after the attempt to extract the tooth, suffering the most excruciating agony, and during all this period being unable to sleep except in a chair.

After the lapse of 5 or 6 months he went to Hot Springs, Ark., where he took baths for about two months and a half; on his return from Hot Springs he had a surgical operation performed for the purpose of removing necrosed bone from the jaw. The surgeon took out several pieces of necrosed bone, the largest being somewhat more than two inches in length, the pieces being jaw bone proper and not alveolus. The largest piece was taken from the portion of the jaw immediately under the second molar, (right side,) and extending two inches forward; this and other pieces were entirely detached from the jaw bone before the operation was performed for their removal. The wounds healed up after the surgical operation by the first intention.

Some two or three months after the attempt at extracting the wisdom tooth, the first and second molar and the second bicuspid on same side became so loose as the result of inflammation, that they had to be removed. The alveolar process under these teeth is all gone.

The patient has a large, healthy, well developed jaw bone and remarkably sound teeth. The wisdom tooth was sound and healthy at the point of fracture.

The plaintiff testified that the defendant made three violent wrenches at his tooth, he each time feeling as though his whole jaw was giving way.

The defendant, that he made but one effort to pull the tooth and then the crown came off with very slight pressure.

The fangs were not taken out of the socket till several months after the attempt to extract the tooth.

ASSUMING THE FACTS TO BE, AS STATED ABOVE,

What was the probable cause of the evils immediately following this accident?

Would those evils have resulted from an operation performed by a dentist possessed of such skill, science an information as to have enabled him properly and judiciously to perform the duties of his calling, if he had used his skill, science and information in performing his operation?"

---

We give place to the above case, because three or four cases somewhat similar have came to our knowledge within the last two years, and in two of them at least, litigation ensued. In one instance at least, the accident occurred in the hands of a dentist of many years experience. Still, long period of practice does not always ensure a large measure of care, nor of professional wisdom, and skill, yet it is presumable that these, to a considerable degree at least, usually all go together.

It is not reasonable to make a diagnosis, or give an opinion especially when the legal rights of any one might be affected thereby, from such a statement as the above. The statement, perhaps is good enough and clear enough so far as it goes, but it is hardly possible that such a statement would reach all the points that should be taken into account. The physician and surgeon, find, in almost every instance of diagnosis, conditions and indications that are indescribable, and yet are important elements in forming a correct opinion; and in almost every case, as in this one, there is conflicting testimony. In this instance, the patient says there were three efforts to extract; the operator says but one. Now at this distance, we are hardly able to determine as to the credibility of these witnesses. The statement of the patient, that he felt as though his whole jaw was giving way, does not carry with it much weight; patients often say that they thought the whole head was giving way, when a tooth is being extracted. In cases where the teeth are firmly set and attached in the sockets, and the wisdom tooth crowds closely upon the ramus of the jaw, the crown may be broken off by the use of the Physick's forceps, and the root remain firmly in place. Great force in the use of this instrument, might break the jaw, especially at or over the age of fifty years. This is too, an unfavorable time life for the repair of ossious fractures.

Physick's forceps is an instrument with which great injury may be done; it is one that should be used but seldom, and then with great care and caution. Safety would dictate that those of little experience should refrain from its use. Extraction of teeth requires for its safe and proper performance, the guidance of thorough anatomical and physiological knowledge, coupled with skillful and careful manipulation.

We are a little apprehensive, that in all cases when severe accidents of this nature occur, there is an absence of some or all of these elements of success.

